# Alternative conformations of a major antigenic site on RSV F

**DOI:** 10.1371/journal.ppat.1007944

**Published:** 2019-07-15

**Authors:** Harrison G. Jones, Michael B. Battles, Chun-Chi Lin, Siro Bianchi, Davide Corti, Jason S. McLellan

**Affiliations:** 1 Department of Biochemistry and Cell Biology, Geisel School of Medicine at Dartmouth, Hanover, New Hampshire, United States of America; 2 Department of Molecular Biosciences, The University of Texas at Austin, Austin, Texas, United States of America; 3 Humabs BioMed SA, a subsidiary of Vir Biotechnology, Bellinzona, Switzerland; Vanderbilt University Medical Center, UNITED STATES

## Abstract

The respiratory syncytial virus (RSV) fusion (F) glycoprotein is a major target of neutralizing antibodies arising from natural infection, and antibodies that specifically bind to the prefusion conformation of RSV F generally demonstrate the greatest neutralization potency. Prefusion-stabilized RSV F variants have been engineered as vaccine antigens, but crystal structures of these variants have revealed conformational differences in a key antigenic site located at the apex of the trimer, referred to as antigenic site Ø. Currently, it is unclear if flexibility in this region is an inherent property of prefusion RSV F or if it is related to inadequate stabilization of site Ø in the engineered variants. Therefore, we set out to investigate the conformational flexibility of antigenic site Ø, as well as the ability of the human immune system to recognize alternative conformations of this site, by determining crystal structures of prefusion RSV F bound to neutralizing human-derived antibodies AM22 and RSD5. Both antibodies bound with high affinity and were specific for the prefusion conformation of RSV F. Crystal structures of the complexes revealed that the antibodies recognized distinct conformations of antigenic site Ø, each diverging at a conserved proline residue located in the middle of an α-helix. These data suggest that antigenic site Ø exists as an ensemble of conformations, with individual antibodies recognizing discrete states. Collectively, these results have implications for the refolding of pneumovirus and paramyxovirus fusion proteins and should inform development of prefusion-stabilized RSV F vaccine candidates.

## Author summary

Respiratory syncytial virus (RSV) is a pervasive pathogen that causes severe lower respiratory tract infections, leading to ~100,000 deaths globally each year and hundreds of millions of dollars in healthcare costs. However, despite its prevalence, there is no vaccine for RSV and the only available therapy is limited to high-risk infants, leaving the vast majority of people with no effective means of prevention or treatment. The RSV fusion (F) protein is a major target of neutralizing antibodies, including extremely potent antibodies that recognize prefusion-specific epitopes. In this study, we determined the crystal structures of two neutralizing human antibodies bound to prefusion RSV F. Our results reveal that each antibody recognizes a different conformation of a neutralization-sensitive epitope, suggesting that this region is inherently flexible and may be important for RSV F function. These results should guide future vaccine-design efforts and help elucidate the mechanisms of RSV F triggering and fusion.

## Introduction

Respiratory syncytial virus (RSV) is a ubiquitous pneumovirus which infects nearly all children in the U.S. by the age of two, with repeated infections occurring throughout life [1]. RSV is a common cause of acute lower respiratory tract infections in young children and the elderly, and in 2015 resulted in an estimated 94,000–149,000 deaths globally in children under the age of five [2]. Although few deaths of children in the United States are attributed to RSV [2, 3], severe infections requiring hospitalization are frequent and lead to estimated direct health care costs of $750 million dollars annually [4]. Currently, there is no vaccine for RSV and the only FDA-approved therapy is passive prophylaxis with the monoclonal antibody palivizumab (Synagis) [5]. However, the high cost and modest efficacy of palivizumab restricts its usage to high-risk infants [6], making the development of improved interventions a global health priority.

RSV is an enveloped virus of the *Pneumoviridae* family and it has a single-stranded, negative-sense RNA genome. There are two subtypes of RSV, A and B, to which many individual strains belong. RSV has two major glycoproteins on the viral surface important for entry: the fusion (F) and attachment (G) glycoproteins [7]. Whereas RSV G is the primary source of sequence variation and defines the subtype of a specific virus, the fusion glycoprotein is well conserved with sequence identities >90% [8]. RSV F is a class I fusion glycoprotein initially produced as an inactive precursor, F0, that is subsequently cleaved by furin-like proteases to generate a protomer of disulfide-linked subunits, F1 and F2 [9–12]. Three of these protomers associate to form the functional trimeric glycoprotein required for membrane fusion and infection [13–15]. Numerous vaccine trials for RSV are currently underway [16], many of which contain the RSV F glycoprotein as an antigen because it has been shown that F is a major target of neutralizing antibodies [17, 18] and is the only protein on the viral surface that is strictly required for entry [19, 20].

RSV F initially folds into a metastable prefusion conformation, with fusion peptides buried within the central cavity of the trimer [8]. During viral entry, RSV F triggers to undergo a dramatic conformational rearrangement from the prefusion to postfusion state. The triggering process results in release of the fusion peptides from the central cavity of the trimer and their insertion into the target-cell membrane, resulting in the formation of an unstable prehairpin intermediate. Collapse of this intermediate into the stable postfusion conformation brings the virus and host-cell membranes together, facilitating formation of a fusion pore and release of the viral genome into the target cell [7, 15]. However, the mechanism and underlying cause of RSV F triggering is not well understood. Recombinant virus expressing only the RSV F protein on its surface is sufficient for infection of immortalized cell lines *in vitro*, suggesting that RSV F can facilitate attachment and mediate fusion in the absence of the attachment glycoprotein [7, 20–22]. Potential RSV F receptors include nucleolin, EGFR, and heparan sulfate proteoglycans, among others [7, 23–27], but the specific role each may play in the setting of natural infection remains to be defined. In addition, *in vitro* experiments have demonstrated that RSV F has a propensity to trigger upon exposure to elevated temperatures [28] and hypo-osmotic conditions [29], and RSV F has even been shown to spontaneously trigger and refold over time due to the metastable nature of the prefusion conformation [30]. This raises the possibility that RSV F does not have a specific receptor that initiates triggering and fusion, but rather that spontaneous triggering in the presence of attachment factors, such as heparan sulfate proteoglycans [31], is sufficient for entry.

The majority of RSV-neutralizing activity in human sera is due to antibodies specific for the prefusion conformation of F [17, 18], and recent characterizations of the human antibody response to RSV F has revealed that prefusion-specific antigenic sites, including site Ø (“zero”), are the major target of neutralizing antibodies [18, 32, 33]. Antigenic site Ø is located at the membrane-distal apex of the trimer and includes the α4-helix and the loop connecting α4 to α5 (α4–α5 loop) of F1, and the F2 loop between β2 and α1. Upon triggering, site Ø undergoes an extensive structural rearrangement in which α4 and the α4–α5 loop refold to form the continuous α5-helix observed in the postfusion F conformation [8]. Comparison of the neutralization potency of two site Ø antibodies, D25 [34] and 5C4 [35], with palivizumab, a site II-directed conformation-independent antibody [36], demonstrated that the prefusion-specific antibodies are 10–100 times more potent [8]. Other potent prefusion-specific human antibodies that bind to the apex of the trimer, such as AM22 and RSD5, have also been isolated in recent years [8, 37, 38], and one of them (MEDI8897) is now in advanced stages of clinical development [39].

D25 was the first structurally characterized antibody that specifically targets prefusion RSV F and was used to solve the structure of the prefusion RSV F conformation, facilitating the engineering of prefusion-stabilized variants that prevent conformational rearrangement to postfusion RSV F [8, 40, 41]. Recently, the structure of 5C4 bound to RSV F was determined, revealing a nearly identical conformation of prefusion RSV F as that observed in the D25-bound structure [42]. However, crystal structures of the different prefusion-stabilized variants of RSV F have revealed an alternative conformation of antigenic site Ø or weak electron density in this region, suggesting that this site is flexible. Currently, it is unclear if flexibility in this region is an inherent property of prefusion RSV F that may be important for triggering membrane fusion [41], or if it is related to inadequate stabilization of site Ø in the engineered variants [40]. Therefore, we sought to investigate the conformational plasticity of site Ø by determining and comparing the crystal structures of prefusion RSV F in complex with AM22 and RSD5. Our results demonstrate that prefusion RSV F adopts at least three alternative conformations of site Ø and that potently neutralizing human antibodies can recognize the alternative conformations using distinct binding modes. This suggests that site Ø samples an ensemble of conformations *in vivo*, at least some of which can be recognized by neutralizing human antibodies. These results should influence future vaccine designs and may have implications for the mechanism of RSV F triggering.

## Results

### AM22 and RSD5-GL bind with high affinity to prefusion RSV F

**Fig 1:**
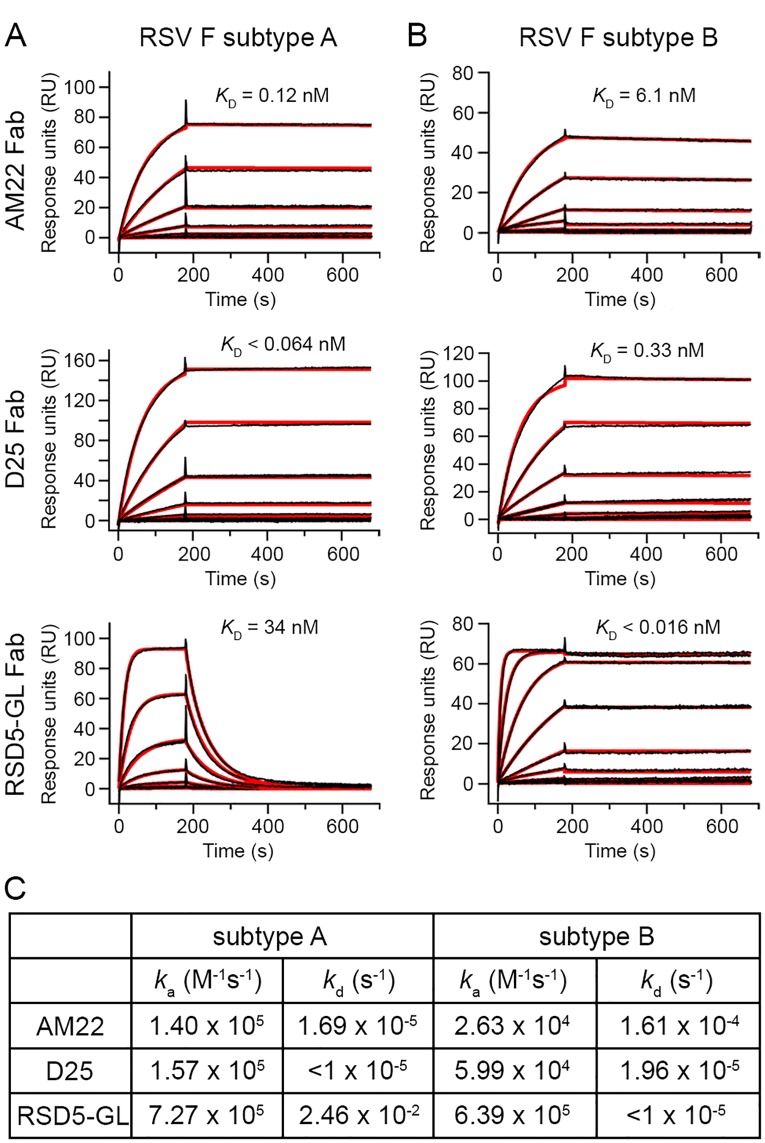
Kinetic parameters of AM22, D25, and RSD5-GL antibodies binding to prefusion RSV F. Surface plasmon resonance (SPR) sensorgrams of AM22, D25, and RSD5-GL Fabs binding to prefusion RSV F from (A) subtype A (strain A2) or (B) subtype B (strain B9320). The raw data is plotted as a black line and the fitted curve used to calculate the binding kinetics is plotted as a thicker red line. Each binding curve has a 180 second association phase, followed by a 500 second dissociation phase. The equilibrium dissociation constant (*K*_D_) is displayed immediately above the SPR curve for each Fab. (C) A table of the association rate constants (*k*_a_) and dissociation rate constants (*k*_d_) determined for the binding kinetics of each Fab. A value of <1 x 10^−5^ was used as the lower limit for the dissociation rate constant (*k*_d_) that can be accurately measured by a Biacore X100.

Previous studies have demonstrated that AM22 and RSD5 potently neutralize RSV and preferentially bind to the prefusion RSV F conformation, similar to the previously characterized site Ø antibodies D25 and 5C4 [8, 35, 38, 42]. However, differences in antibody kinetics and subtype specificities have not been fully explored. Therefore, we used surface plasmon resonance (SPR) to determine the binding affinity and kinetics of the interaction between three site Ø antibodies (AM22, D25, and RSD5) and prefusion RSV F derived from each subtype (strains A2 and B9320) (Fig 1). For these studies, we worked with a germline-reverted version of RSD5 (RSD5-GL), which had 20 somatic mutations in the framework regions reverted to germline residues to minimize immunogenicity (S1 Fig). Of note, RSD5-GL showed similar neutralization potency and binding kinetics for prefusion RSV F as compared to the parental RSD5 antibody (RSD5-WT) (S2 Fig). Despite similar neutralization potencies, AM22, D25, and RSD5 displayed distinct affinities and binding kinetics when compared to each other as well as when compared individually across the two RSV subtypes. The AM22 antigen-binding fragment (Fab) has an ~50-fold higher affinity for subtype A with an equilibrium dissociation constant (*K*_D_) of 0.12 nM, whereas its affinity for subtype B is 6.1 nM. Similarly, D25 Fab binds tightly to prefusion RSV F with a slight preference for subtype A, consistent with previously published data [43], having a *K*_D_ of <0.064 nM and 0.33 nM for subtype A and B, respectively. In contrast, RSD5-GL Fab has substantial subtype specificity with a >2,000-fold stronger affinity for subtype B compared to subtype A, with a *K*_D_ of <0.016 nM and 34 nM, respectively. Preferential binding of RSD5-GL for subtype B is primarily due to the off-rate, which is fast for the subtype A interaction and slow for the subtype B interaction.

### Structure determination of AM22 in complex with prefusion RSV F

To investigate the conformation of site Ø and define the epitope on prefusion RSV F recognized by AM22, we determined the crystal structure of the AM22 Fab alone and in complex with the prefusion-stabilized RSV F variant DS-Cav1 [40]. Crystals of the AM22 Fab alone diffracted X-rays to 1.7 Å and crystals of the prefusion RSV F–AM22 complex diffracted X-rays to 3.5 Å (Table 1). The AM22 variable domain (Fv) superimposes very well between the bound and unbound crystal structures, with high structural similarity across the framework and complementarity-determining regions (CDRs) resulting in an r.m.s.d. of ~0.31 Å for 214 Cα atoms.

**Table 1:** Crystallographic data collection and refinement statistics.

	AM22 Fab	DS–Cav1 + AM22 Fab	DS–Cav1 + RSD5-GL Fab
**PDB ID**	6DC4	6DC5	6DC3
**Data collection**			
Space group	*P*2_1_2_1_2_1_	*P*2_1_22_1_	*I*23
Wavelength (Å)	1.000	1.000	0.979
Cell dimensions			
*a*, *b*, *c* (Å)	64.2, 75.2, 109.0	132.4, 152.2, 202.9	270.3, 270.3, 270.3
α = β = γ (°)	90	90	90
Resolution (Å)	32.7–1.7 (1.73–1.70)*	50.5–3.5 (3.61–3.50)	38.2–3.5 (3.64–3.50)
*R*_merge_	0.069 (0.739)	0.313 (0.903)	0.094 (0.755)
*I* / σ*I*	17.4 (2.3)	5.7 (2.0)	9.7 (1.8)
CC_1/2_	0.999 (0.714)	0.933 (0.363)	0.995 (0.528)
Completeness (%)	100 (100)	99.8 (98.6)	99.9 (100)
Redundancy	7.1 (6.5)	5.6 (5.0)	4.8 (4.6)
Total reflections	419,220 (20,172)	292,867 (21,953)	198,500 (21,266)
Unique reflections	58,782 (3,082)	52,333 (4,425)	41,327 (4,656)
**Refinement**			
Resolution (Å)	32.7–1.7 (1.74–1.70)	47.2–3.5 (3.56–3.50)	38.2–3.5 (3.59–3.50)
Unique reflections	58,706 (4,114)	52,294 (2,685)	41,305 (2,925)
*R*_work_ / *R*_free_ (%)	18.0/20.5	21.7/28.0	18.3/20.5
No. atoms	3,955	20,286	6,890
Protein	3,291	20,199	6,797
Water	596	-	-
NAG	-	42	28
PEG	-	42	-
Cd^2+^	-	3	-
SO_4_^2-^	-	-	65
EDO	68	-	-
*B*-factors (Å^2^)			
Protein	19.8	65.7	131.3
Water	32.2	-	-
NAG	-	55.5	200.2
PEG	-	21.8	-
Cd^2+^	-	109.4	-
SO_4_^2-^	-	-	200.7
EDO	29.0	-	-
R.m.s. deviations			
Bond lengths (Å)	0.006	0.003	0.002
Bond angles (°)	0.84	0.64	0.59
Ramachandran (%)			
Favored	98.1	95.3	95.4
Allowed	1.9	4.6	4.4
Outliers	0	0.1	0.2

Data were collected from one crystal.

*Values in parentheses are for highest-resolution shell.

**Fig 2:**
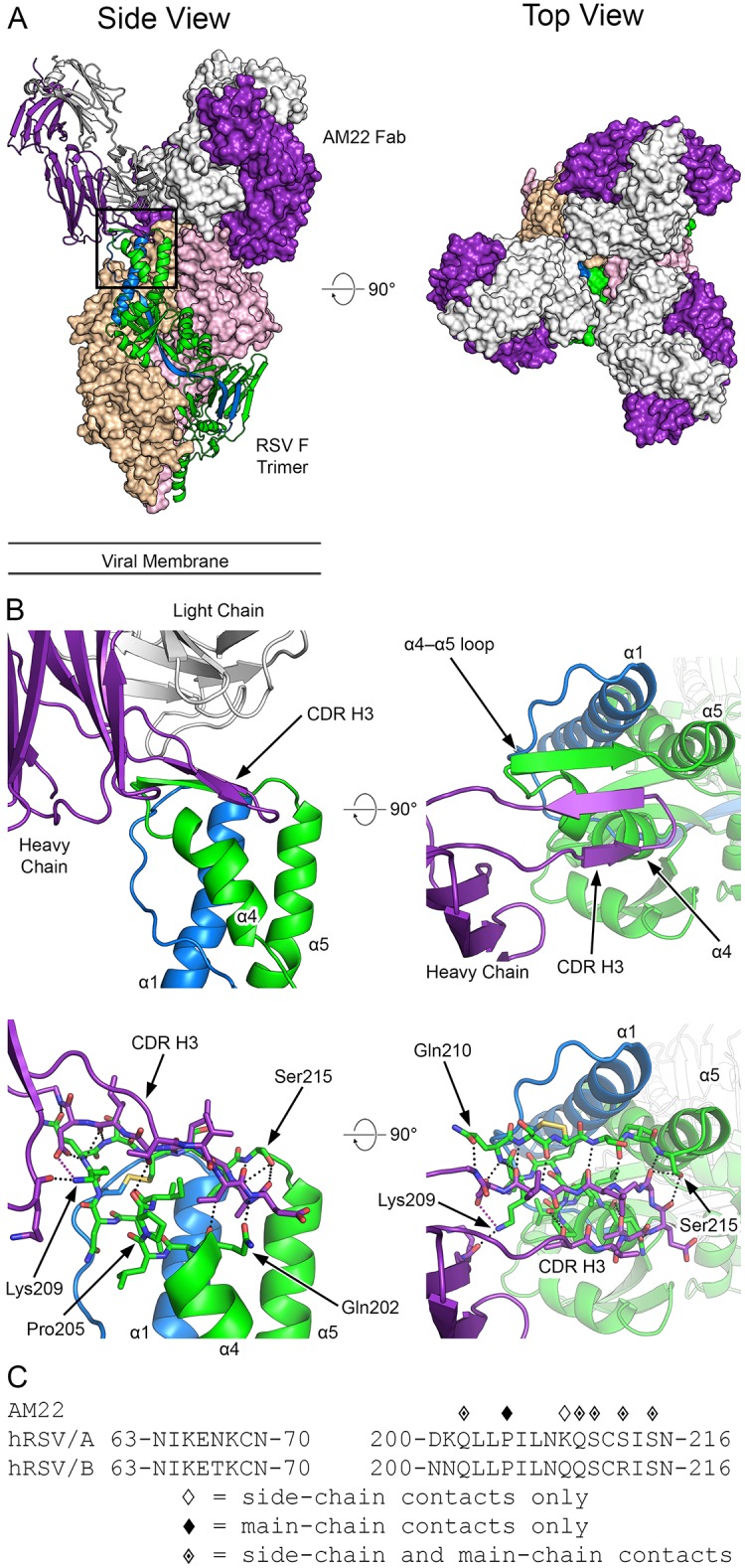
Structure of AM22 in complex with prefusion RSV F. (A) Crystal structure overview shows three AM22 Fabs bound to the prefusion RSV F trimer, viewed looking along or toward the viral membrane. One RSV F protomer and one AM22 Fab are shown in ribbon representation (left panel), whereas the other protomers and Fabs are shown as molecular surfaces. The AM22 heavy chain is colored purple and the light chain is white. One RSV F protomer is colored green for F1 and blue for F2, whereas the other two RSV F protomers are tan and pink. (B) Close-up of the side and top views in ribbon-and-stick representation, colored as in (A), highlighting the β-sheet hydrogen bond interactions between F1 and the CDR H3 of AM22. The light chain was hidden in the lower-left panel and both panels on the right for clarity. For stick models, oxygen atoms are colored red, nitrogen blue, and sulfur yellow. (C) The amino acid sequence of RSV F site Ø is shown for both strain A2 and strain B9320. Diamond symbols above each residue indicate a contact between AM22 and prefusion RSV F strain A2 based upon PDBePISA analysis of the crystal structure.

The crystal structure of the F–AM22 complex shows that three AM22 Fabs bind to prefusion RSV F at the membrane-distal apex of the trimer and have a vertical angle of approach (Fig 2A), in agreement with previously published negative stain EM images [8]. AM22 buries 729 Å^2^ of surface area on each protomer of prefusion RSV F, mediated primarily through interactions between the heavy chain and F1 subunit.

The AM22 heavy chain buries 554 Å^2^ (76%) on the surface of prefusion RSV F and is involved in 15 hydrogen bonds with RSV F, 14 of which are formed between the CDR H3 and seven residues within Gln202–Ser215 of α4 and the α4–α5 loop of prefusion RSV F. The light chain is responsible for the remaining 175 Å^2^ (24%) of buried surface area on prefusion RSV F and forms three additional hydrogen bonds with the α4–α5 loop via the CDR L2. The high affinity and specificity of AM22 for prefusion RSV F (Fig 1 and S3 Fig) is due to the formation of a three-strand anti-parallel β-sheet between the CDR H3 of AM22 and α4–α5 loop of F1 (Fig 2B). When bound by AM22, the α4-helix kinks near residue Pro205 and shifts away from α5, stretching the α4–α5 loop and allowing it to adopt a β-strand conformation that pairs with the β-hairpin formed by the CDR H3 of AM22. Upon RSV F triggering and the rearrangement into the postfusion conformation, α4 and the α4–α5 loop refold into the continuous α5-helix, which would disrupt the β-sheet interaction and prevent AM22 binding.

Sequence comparison of the two RSV F subtypes demonstrates that the residues comprising the AM22 epitope are well-conserved. However, one of the subtype A RSV F residues that contacts AM22 is Lys209, which is a Gln in subtype B (Fig 2C). The Lys209 side chain of subtype A prefusion RSV F is coordinated by three residues of the AM22 heavy chain. This includes the formation of a salt bridge with Asp100G of the CDR H3 that effectively extends the prominent β-sheet interaction. Substitution of Lys209 with Gln, as found in subtype B, would eliminate the salt bridge and may explain the subtype A preference of AM22. Indeed, incorporating the K209Q substitution in subtype A prefusion RSV F results in a decreased affinity of AM22 with a *K*_D_ of 6.7 nM (S4 Fig), which closely matches the *K*_D_ of 6.1 nM for subtype B.

### Crystal structure of RSD5-GL in complex with prefusion RSV F

**Fig 3:**
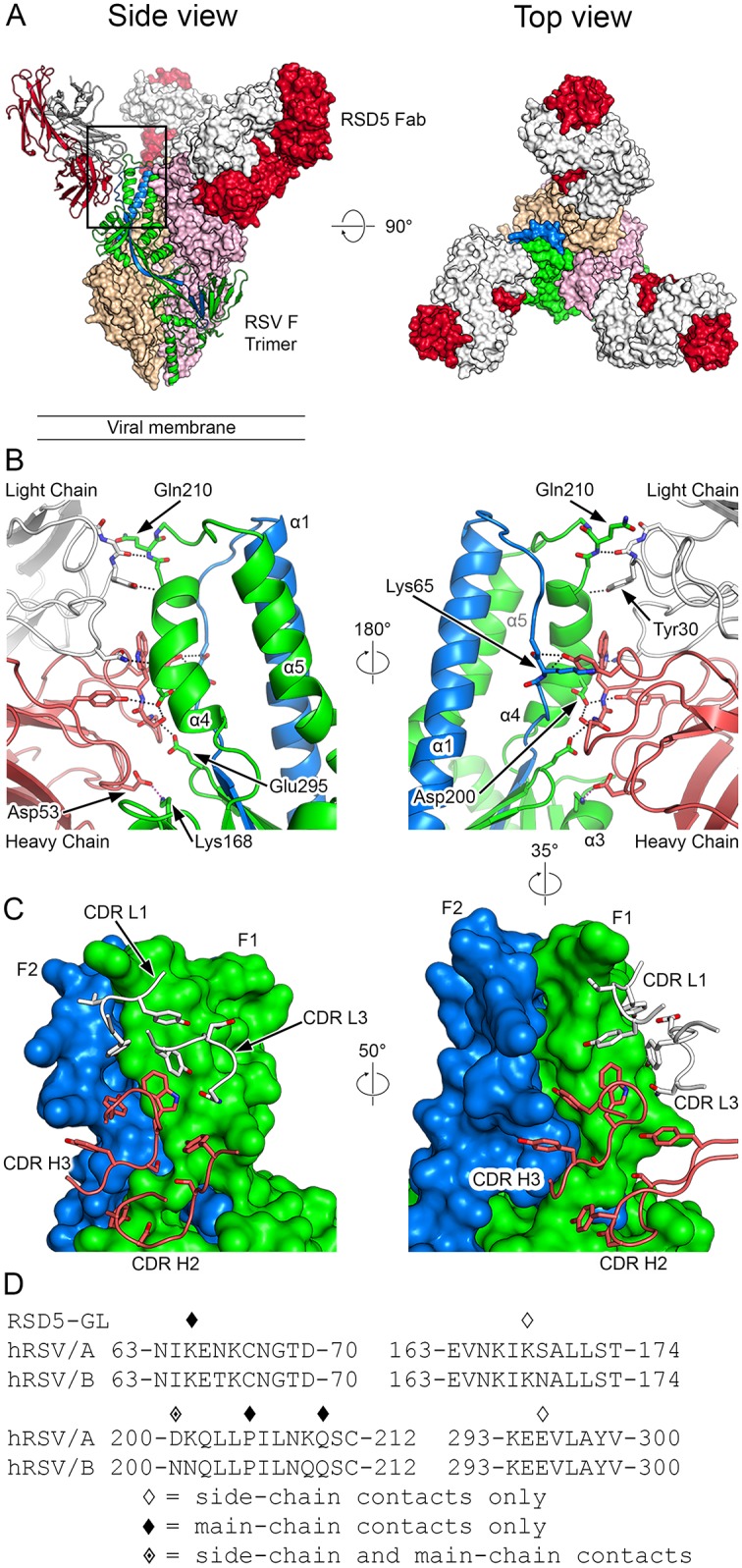
Structure of RSD5-GL in complex with prefusion RSV F. (A) Crystal structure overview shows three RSD5-GL Fabs bound to the prefusion RSV F trimer, viewed looking along or toward the viral membrane. One RSV F protomer and the contacted RSD5-GL Fab are shown in ribbon representation (left panel), whereas the other protomers and Fabs are shown as molecular surfaces. The RSD5-GL heavy chain is colored red and the light chain is white. One RSV F protomer is colored green for F1 and blue for F2, whereas the other two RSV F protomers are tan and pink. (B) Close-up of the side views of the interactions between one RSD5-GL Fab and one RSV F protomer in ribbon-and-stick representation, rotated 180° from each other and colored as in (A). For stick models, oxygen atoms are colored red, nitrogen blue, and sulfur yellow. (C) Ribbon-and-stick model of the RSD5-GL CDR loops contacting one protomer of RSV F, which is shown as a molecular surface. Colored as in (B) and rotated as indicated. (D) The amino acid sequence of residues near RSV F site Ø and site V are shown for both strain A2 and strain B9320. Diamond symbols above each residue indicate a contact between RSD5-GL and prefusion RSV F strain A2 based upon PDBePISA analysis of the crystal structure.

To further investigate the conformational variability of site Ø and to identify the epitope on prefusion RSV F recognized by RSD5-GL, we determined the crystal structure of prefusion RSV F in complex with the RSD5-GL Fab to 3.5 Å resolution (Table 1). The crystal structure shows that three RSD5-GL Fabs bind to the membrane-distal apex of the prefusion RSV F trimer (Fig 3A). RSD5-GL binds slightly lower on the trimer than AM22, bridging antigenic site Ø and the recently defined site V [32, 44]. The interactions of RSD5-GL with prefusion RSV F are more diverse than that of AM22, with the CDR H2 and CDR H3 as well as all three CDRs of the light chain making contacts with the F protein (Fig 3B). In addition, the contacts on prefusion RSV F span multiple regions including the F2 loop as well as α3 and α4 of F1, burying a total surface area of 855 Å^2^.

The RSD5-GL heavy chain buries 577 Å^2^ (67%) on the surface of prefusion RSV F. The CDR H3 interacts with α4 and the F2 loop, forming hydrogen bonds with Asp200 and main-chain atoms of Lys65, respectively, whereas the CDR H2 contacts α3 and forms a salt bridge with Lys168. The light chain contributes to the interface through contacts with α4, including hydrogen bonds with Asp200 and main-chain atoms of Pro205 and Gln210. Similar to AM22, the prefusion specificity of RSD5-GL can be explained by the dramatic rearrangement of α3 and α4 to form the single elongated α5-helix upon conversion to the postfusion conformation, which dismantles the RSD5-GL epitope.

### Structural comparison of the binding modes of site Ø antibodies

**Fig 4:**
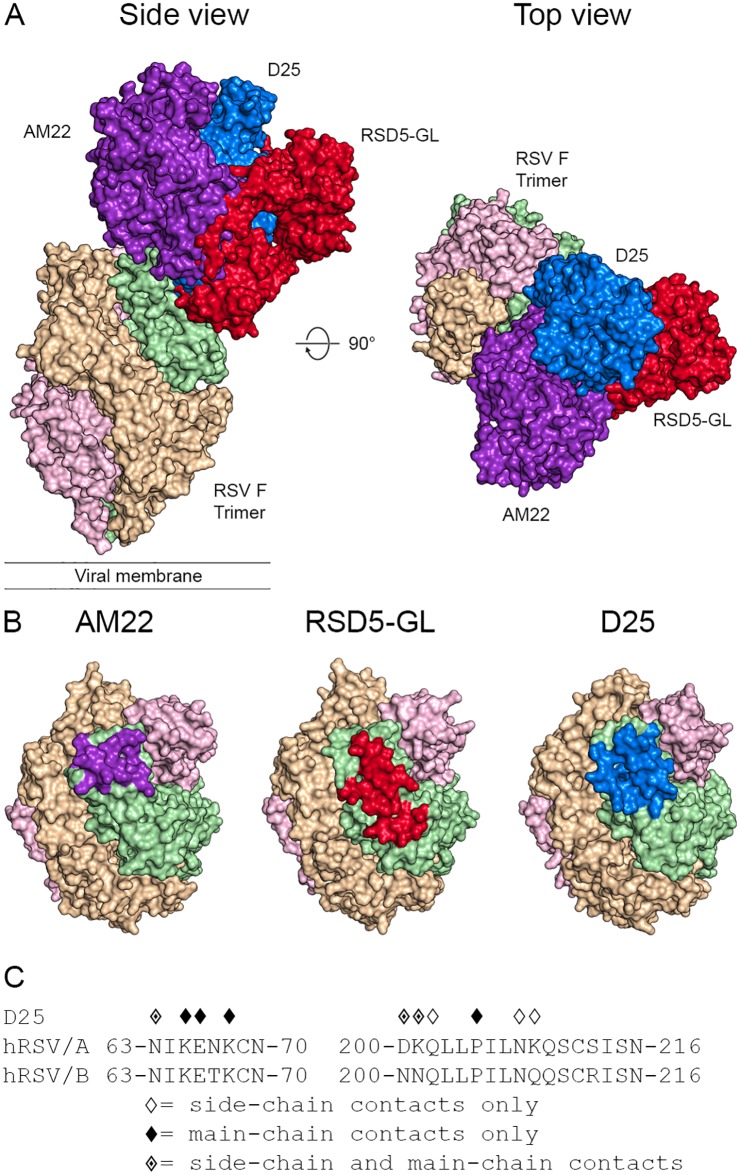
AM22, RSD5-GL, and D25 have overlapping epitopes at the apex of the RSV F trimer. (A) Surface representations of a single AM22, RSD5-GL, and D25 Fab bound to the apex of the RSV F trimer, viewed looking along or toward the viral membrane. The three RSV F protomers are colored light green, tan, and pink. AM22 is colored purple, RSD5-GL is colored red, and D25 is colored blue. (B) Surface representations of the apex of the RSV F trimer highlight the epitope of each antibody on a single F protomer. The epitope for each antibody is shown on the green protomer, and the epitope itself is colored purple for AM22, red for RSD5-GL, and blue for D25. (C) The amino acid sequence of residues near RSV F site Ø is shown for both strain A2 and strain B9320. Diamond symbols above each residue indicate a contact between D25 and prefusion RSV F strain A2 based upon PDBePISA analysis of the crystal structure.

The binding mode and angle of approach differs for each of the three site Ø antibodies (Fig 4). AM22 and D25 adopt a vertical angle of approach and bind at the apex of the trimer, in agreement with previous negative stain EM images [8, 42]. In contrast, RSD5-GL has a more diagonal angle of approach and binds slightly lower on prefusion RSV F, bridging antigenic sites Ø and V, similar to the recently characterized antibody 5C4 [42]. Despite these differences, the epitopes of all three antibodies overlap significantly and large steric clashes would prevent any two of these antibodies from binding simultaneously (Fig 4A). Specifically, all three antibodies make multiple competing contacts with α4 and the α4–α5 loop.

In addition, the extent to which the antibodies interact with the F2 subunit varies greatly between the three antibodies. The RSD5-GL interface with F2 accounts for 21% of the buried surface area on prefusion RSV F and includes two hydrogen bonds with Lys65. The D25 interface with F2 contributes 23% of the buried surface area and includes five hydrogen bonds to four residues within Asn63–Lys68. In contrast, the interface between AM22 and F2 accounts for only 9% of the buried surface area on prefusion RSV F, and AM22 forms no hydrogen bonds or salt bridges with F2. Thus, whereas RSD5-GL and D25 make several contacts with the F2 loop, AM22 interacts almost exclusively with the F1 subunit.

### Varying conformations of antigenic site Ø indicate a natural flexibility of prefusion RSV F

**Fig 5:**
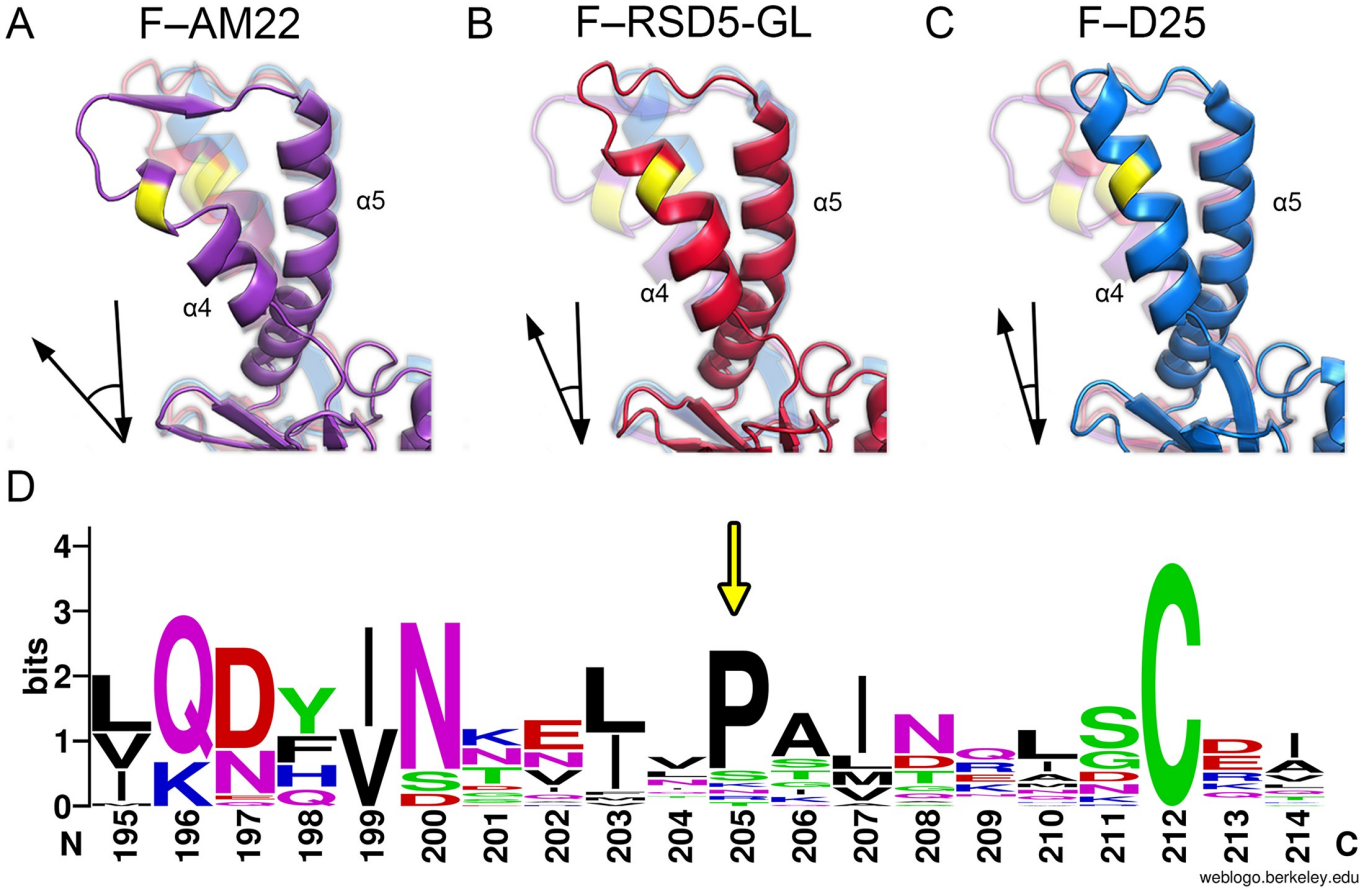
Alternative conformations of site Ø. (A–C) Ribbon representation of prefusion RSV F from the three indicated antibody complexes. The alternative conformations structurally diverge near proline 205 (colored yellow). Each panel (A–C) also contains faded versions of the two other conformations for direct comparison. Arrows in the bottom left corner of each panel depict the angle between α4 and α5 of the conformation displayed in that panel. (D) A WebLogo generated from representative sequences of all known pneumovirus and paramyxovirus fusion proteins corresponding to the α4-helix and α4–α5 loop indicates a well-conserved proline (indicated by the yellow arrow). Residue numbering is based upon the human RSV fusion protein sequence from strain A2.

Alignment of the three antibody-bound prefusion RSV F structures revealed three alternative conformations of site Ø (Fig 5). The structure of DS-Cav1 in complex with AM22 shows a more open conformation of this site, with the α4-helix kinked out and away from α5, resulting in a stretched α4–α5 loop and a larger angle between the α4 and α5 helices. This structure closely matches the unbound prefusion-stabilized RSV F variants DS-Cav1 (PDB ID: 4MMU) and PR-DM (PDB ID: 5C69 [41]), specifically the kink in the α4-helix and greater angle between α4 and α5. In contrast, the prefusion RSV F–D25 structure has a more closed conformation of site Ø, where α4 does not kink out and there is a smaller angle between α4 and α5. The structure of DS-Cav1 bound to RSD5-GL reveals an intermediate site Ø conformation, with the α4-helix only slightly kinked out and away from α5, but not to the same degree as seen in the AM22 complex. Because all three of these antibodies were isolated from humans who had experienced natural RSV infection, these structures indicate that site Ø is naturally flexible and adopts at least three states that can be recognized by the human immune system. It is also possible, and perhaps more likely, that site Ø exists as an ensemble of many conformations, three of which were trapped by these antibodies. Analysis of published crystal structures of prefusion RSV F in complex with neutralizing antibodies targeting various antigenic sites further supports an ensemble of site Ø conformations (S5 Fig).

## Discussion

AM22 and RSD5 are two human antibodies that bind to antigenic site Ø and are specific for the prefusion conformation of RSV F. Although both antibodies potently neutralize RSV, similar to D25, their binding kinetics and subtype specificity have distinct differences. AM22 and D25 both preferentially bind to subtype A, whereas RSD5 preferentially binds to subtype B as a result of its fast dissociation rate constant for subtype A F proteins. In addition, previous publications using SPR and flow cytometry-based competition assays have suggested that AM22 and RSD5 do not compete with D25, but rather occupy a separate prefusion-specific epitope [37, 38]. However, comparing the crystal structures of prefusion RSV F bound to AM22, RSD5-GL, and D25, demonstrates that all three antibody epitopes overlap substantially and would prevent any two from binding simultaneously due to large steric clashes. This emphasizes the importance of structural characterization of antibody epitopes in addition to competition data, as varying antibody kinetics can mislead epitope classification when using only competition assays.

The structural studies presented here reveal alternative conformations of RSV F site Ø in the prefusion state and suggest a natural flexibility of the region that can be recognized in numerous ways by the humoral immune system. This conformational flexibility is further supported by crystal structures of multiple prefusion-stabilized variants, which have identified an array of site Ø conformations, very high atomic B factors, or a distinct lack of site Ø electron density (PDB IDs: 4JHW, 4MMQ–4MMV, 4ZYP, 5C69, 5C6B, 5EA3–5EA8, 5KWW, 5K6B, 5K6C, 5K6F–5K6I, 5TOJ, and 5TOK [8, 40, 41, 45–49]). Conformational flexibility and transient exposure of different epitopes have been noted for other class I fusion proteins such as HIV-1 Env and MERS-CoV Spike [50–54]. Specifically, identical residues of the V1/V2 loops of HIV-1 Env have been shown to adopt different conformations when bound by two different neutralizing antibodies, demonstrating structural plasticity of an important neutralizing epitope [55, 56].

Comparison of two prefusion-stabilized RSV F variants, DS (PDB ID: 4MMQ) and Cav1 (PDB ID: 4MMS), highlights the flexibility of site Ø and suggests that a conformational rearrangement of site Ø is required prior to release of the fusion peptide from the central cavity of the trimer during refolding from the prefusion to postfusion state [40]. When only the fusion peptide was anchored by a disulfide bond in the DS structure (PDB ID: 4MMQ), site Ø was conformationally heterogeneous as indicated by the lack of electron density. However, site Ø cavity-filling mutations introduced in the Cav1 (PDB ID: 4MMS) or DS-Cav1 (PDB ID: 4MMU) variants stabilized the prefusion RSV F structure and showed clear electron density extending from the fusion peptide through site Ø. Taken together, this suggests that movement of the fusion peptide is conditional upon prior rearrangement of site Ø. The only discrepancy between the crystal structures of wild-type prefusion RSV F when bound to D25 and the prefusion-stabilized variant DS-Cav1 is the kinked-open conformation of α4 in the DS-Cav1 model, which was attributed to the cavity-filling V207L mutation being unable to fully stabilize the α4-helix [40]. However, the structure reported here of DS-Cav1 in complex with the human antibody AM22 matches the unbound DS-Cav1 structure and demonstrates that this conformation is a naturally sampled alternative conformation of prefusion RSV F.

Recent vaccine strategies targeting viruses with class I fusion proteins have focused on stabilizing the prefusion conformation of the fusion protein for use as an immunogen. A common approach to achieve prefusion stabilization has been through introduction of one or more proline residues within the loop of a helix-loop-helix motif that refolds into a continuous alpha helix in the postfusion conformation. This strategy has been used successfully to stabilize several class I viral fusion glycoproteins including RSV F, MPV F, influenza HA, HIV Env, and coronavirus Spike [41, 57–60]. Proline-based stabilization utilizes the restricted phi-psi angles of proline residues and disruption of the alpha-helix hydrogen bond network to inhibit the hinge motion required of the helix-loop-helix motif and the subsequent coil-to-helix structural transition required for refolding into the postfusion conformation. However, for wild-type prefusion RSV F there is a conserved proline residue (Pro205) within the middle of the α4-helix, N-terminal to the α4–α5 loop that may act as a hinge during refolding [41]. Crystal structures of prefusion RSV F demonstrate a variety of alternate conformations adopted by antigenic site Ø, all of which diverge near the conserved Pro205. This indicates that Pro205 may disfavor a rigid alpha-helical structure, which in turn facilitates conformational flexibility of site Ø and the tendency of prefusion RSV F to trigger. We note that Pro205 is absolutely conserved in all human and bovine RSV strains, and a proline at an identical position is also commonly found in F proteins from viruses within the *Pneumoviridae* and *Paramyxoviridae* families, with the exception of human metapneumoviruses (Fig 5D). The functional importance of this well-conserved proline residue will need to be evaluated in future studies investigating the triggering mechanism and refolding of pneumovirus and paramyxovirus F proteins.

Recent characterization of the antibody repertoire against RSV F highlights the importance of prefusion-specific epitopes when selecting immunogens for RSV F vaccine design, particularly after the recent failure of several postfusion RSV F vaccine trials [16, 18, 32, 33, 61, 62]. Our findings demonstrate that potently neutralizing human antibodies that target antigenic site Ø can recognize a variety of alternative conformations and have varying degrees of subtype specificity. Because potent antibodies can bind to the various alternative conformations of site Ø, we propose that the ideal prefusion RSV F immunogen would maintain this natural conformational flexibility of site Ø as well as present neutralizing epitopes common to both RSV subtypes.

## Methods

### Viruses

The RSV viruses used in this study were A/A2/61 (ACO83301), A/Randall/58, A/Long/56 (ACO83302), B/WV/14617/85 (ATCC VR-1400), A/9320/77 (AAR14266), A/9846/09 (JX171066), A/9835/09 (JX171067), A/9278/07 (KC618407), A/9395/07 (KC618409), B/9736/09 (JX171068), B/9847/09 (JX171073).

### Production of RSV F proteins

Plasmid encoding prefusion-stabilized RSV F (DS-Cav1), subtype A DS-Cav1 with a K209Q substitution, or postfusion RSV F based on subtype A (strain A2) or subtype B (strain B9320) with a C-terminal 6x- or 8x-histidine tag and Strep-tag II was co-transfected with furin into FreeStyle 293-F cells (Invitrogen) at a 4:1 ratio to ensure full cleavage of prefusion RSV F. Proteins were purified from the media after six days using Ni-NTA Superflow resin (Qiagen) and *Strep*-Tactin resin (IBA). Tags were removed by digestion with thrombin protease, followed by gel filtration using a Superdex 200 16–600 column (GE Healthcare Biosciences). Prefusion RSV F protein used for SPR was produced in the same manner, except the tags were not removed prior to gel filtration. For crystallization, DS-Cav1 from strain A2 was expressed in the presence of kifunensine (5 μM), digested with 10% (*w/w*) Endo H overnight, mixed with a 2-fold or 1.5-fold molar excess of purified Fab for the AM22–RSV F and RSD5-GL–RSV F complexes, respectively, and the resulting complexes were purified by size exclusion chromatography (SEC) using the Superose 6 XK 16–70 column (GE Healthcare Biosciences) in a buffer consisting of 2 mM Tris pH 8.0, 200 mM NaCl, and 0.02% NaN_3_.

### Production of AM22, RSD5, and D25 IgGs and Fabs

Germline sequences of RSD5 framework regions were determined with reference to the IMGT database [63]. RSD5 and RSD5-GL (fully germlined in VH and VL framework regions, as defined by IMGT) were produced by gene synthesis (GenScript) and confirmed by sequencing. Synthesized VH and VL sequences were cloned into human Igγ1 and Igκ expression vectors (kindly provided by Michel Nussenzweig, Rockefeller University, New York, NY, USA), essentially as described [64]. Plasmids encoding antibody heavy and light chains for AM22, RSD5-WT, RSD5-GL, or for D25 were co-transfected into Expi293 cells or FreeStyle 293-F cells (Invitrogen). AM22 and D25 IgGs and Fabs were purified using Protein A agarose (Fisher) or CaptureSelect IgG-CH1 affinity matrix (Life Technologies), respectively. All IgG antibodies were eluted off the Protein A column using 0.1 M glycine pH 3.0 into a buffered solution containing 1/10 (*v/v*) of 1 M Tris pH 8.0. All Fabs were eluted off the CaptureSelect IgG-CH1 column using 50 mM acetic acid pH 4.0 into a buffered solution containing 1/10 (*v/v*) of 1 M Tris pH 8.0. To produce RSD5-WT Fab, RSD5-WT was expressed and purified as an IgG with an HRV 3C protease site in the hinge of the heavy chain. RSD5-WT Fab was produced by digesting the IgG with HRV 3C for 2 hours at room temperature, followed by passing the solution over protein A resin to remove the Fc, and subsequently purified by SEC using a Superdex 200 column (GE). Production of RSD5-GL Fab was done by incubating RSD5-GL IgG with papain beads (Pierce). All IgGs and Fabs were buffer exchanged using a desalting column, followed by final purification by SEC using a Superdex 200 column (GE) prior to long term storage at -80 °C.

### Surface plasmon resonance

DS-Cav1 or postfusion RSV F from subtype A (strain A2) or subtype B (strain B9320), as well as a subtype A mutant with a K209Q substitution, with a C-terminal 6x-His or 8x-His tag was immobilized on a Ni-NTA sensor chip to a total of 80–150 response units using a Biacore X100 (GE). A buffer-only sample was injected over the DS-Cav1 or postfusion RSV F and reference flow cells for reference subtraction, followed by serial 3-fold dilutions of Fab (AM22, RSD5-GL, RSD5-WT, or D25) from 300 nM to 46.5 pM in HBS-P+, with a duplication of the 11.1 nM concentration. For the DS-Cav1 subtype A mutant with a K209Q substitution, only AM22 Fab was evaluated. For the assay evaluating AM22 Fab binding to strain B9320, the highest AM22 Fab concentration used was 1 uM in HBS-P+ buffer, followed by serial 3-fold dilutions to the lowest concentration of 152 pM. For the assay evaluating binding to postfusion RSV F of subtype A or B, a concentration of 300 nM of each Fab in HBS-P+ buffer was used. The data were double-reference subtracted and fit to a 1:1 binding model using the Biacore X100 or Scrubber2 analysis software. Final binding curves were displayed using GraphPad Prism Version 7.03 for Windows.

### Virus neutralization

Microneutralization assay based on infection of Hep-2 cells by RSV strains. RSD5 human IgG1 monoclonal antibody variants were incubated with 50–100 TCID_50_ of viruses for 1 hour at room temperature before addition of Hep-2 target cells which were incubated for 4 or 5 days (depending on the strain). Viral infection was measured by indirect immunofluorescence using an automated Pathway 855 analyzer (BD) as previously described [38]. IC_50_ values were calculated by interpolation of neutralization curves fitted with a 4-parameter nonlinear regression with a variable slope.

### Crystallization and data collection

Crystals for the AM22 Fab alone were produced by sitting-drop vapor diffusion using the Morpheus HT-96 crystallization screen (Molecular Dimensions). AM22 Fab (18.0 mg/mL in 100 mM NaCl, 1 mM tris pH 8.0, 0.01% NaN_3_) was mixed at a 1:2 ratio with the D2 reservoir condition (0.02 M 1,6-hexanediol; 0.02 M 1-butanol; 0.02 M 1,2-propanediol (racemic); 0.02 M 2-propanol; 0.02 M 1,4-butanediol; 0.02 M 1,3-propanediol; 0.1 M MES/imidazole pH 6.5; 10% (*w/v*) PEG 8,000; 20% ethylene glycol). Crystals were looped directly from the crystallization drop and frozen in liquid nitrogen. Data were collected at the X6A beamline (National Synchroton Light Source, Brookhaven National Laboratories) and scaled to 1.70 Å.

The best diffracting crystals of the complex of Endo H-treated DS-Cav1 with AM22 Fab were produced using the Hampton HT Additive Screen via sitting-drop vapor diffusion. We mixed 100 nL of DS-Cav1–AM22 (5.7 mg/mL in 200 mM NaCl, 2 mM Tris pH 8.0, 0.02% NaN_3_) with 200 nL of reservoir solution containing 0.1 M sodium acetate pH 5.5, 32.15% (*v/v*) PEG 400, 4.02% (*w/v*) PEG3350, and 0.01 M cadmium chloride. Crystals were looped directly from the crystallization drop and frozen in liquid nitrogen. Data were collected at the X6A beamline (National Synchroton Light Source, Brookhaven National Laboratories) and scaled to 3.50 Å.

Crystals for the complex of Endo H-treated DS-Cav1 with RSD5-GL Fab were initially identified in position H3 of the ProPlex HT-96 crystallization screen (Molecular Dimensions) via sitting-drop vapor diffusion. The best diffracting crystal was grown in a solution of 1.8 M lithium sulfate and 0.1 M Tris at pH 8.0 via hanging-drop vapor diffusion at a protein-to-reservoir ratio of 1:3 by mixing 0.5 μL of DS-Cav1–RSD5-GL (5.9 mg/mL) with 1.5 μL of reservoir solution. The crystal was looped directly from the crystallization drop and flash frozen in liquid nitrogen. X-ray diffraction data for this complex were collected at the 19-ID beamline (Advanced Photon Source, Argonne National Laboratories) and scaled to 3.50 Å.

### Structure determination, model building, refinement, and analysis

Diffraction data were processed using the CCP4 software suite [65]: data were indexed and integrated in iMOSFLM [66] and scaled and merged with AIMLESS [67]. A molecular replacement solution for the 1.70 Å AM22 Fab dataset was found by PHASER [68] using a chimeric protein model consisting of the heavy and light chains of PDB ID: 3LMJ and PDB ID: 3QEG, respectively, separated into the constant and variable domains as search models. The structure was built manually in Coot [69] and refined using PHENIX [70]. The structure was built and refined to an *R*_work_/*R*_free_ of 18.0%/20.5% (Table 1).

A molecular replacement solution for the 3.50 Å complex of DS-Cav1 with AM22 Fab was obtained using PHASER with prefusion-stabilized RSV F variant Cav1 (PDB ID: 4MMS) and the 1.70 Å AM22 Fab structures as search models. The asymmetric unit contained the prefusion trimer bound by three AM22 Fabs. Rigid-body refinement was then performed in PHENIX, followed by refining group B-factors and (x, y, z) coordinates in PHENIX with NCS torsion restraints and reference-model restraints. The reference model was the 2.40 Å prefusion-stabilized RSV F variant Cav1 (PDB ID: 4MMS). The structure was built and refined to an *R*_work_/*R*_free_ of 21.7%/28.0% (Table 1).

A molecular replacement solution for the 3.50 Å complex of DS-Cav1 with RSD5-GL Fab was obtained using PHASER with prefusion-stabilized RSV F variant PR-DM (PDB ID: 5C69) and a chimeric protein Fab model consisting of the heavy and light chains of PDB ID: 1DFB and PDB ID: 1MCO, respectively, separated into the constant and variable domains and without the Fc domain of the 1MCO heavy chain. The structure was built manually in Coot [69] and refined using PHENIX [70]. Rigid-body refinement was initially performed in PHENIX, followed by refining individual B-factors and (x, y, z) coordinates in PHENIX with reference-model restraints. The reference model was the 2.3 Å prefusion F variant PR-DM (PDB ID: 5C69). The structure was built and refined to an *R*_work_/*R*_free_ of 18.3%/20.5% (Table 1).

Structural features were analyzed using the “Interfaces” feature of PDBePISA [71]. This analysis defined the antibody epitope and paratope, specific residues and contacts involved in the interface, as well as the buried surface area. The modeled structure of each complex was displayed using PyMOL [72] to facilitate structural comparison between the different complexes.

### Sequence analysis of *Pneumoviridae* and *Paramyxoviridae* fusion proteins

The amino acid sequence for the human respiratory syncytial virus subtype A (strain A2) fusion protein was used as the original sequence for comparison with all known pneumovirus and paramyxovirus fusion proteins. NCBI basic local alignment search tool (BLAST) was used to identify homologous regions between the hRSV fusion protein sequence (strain A2) and other pneumovirus and paramyxovirus fusion proteins. Specifically, we identified sequences that were indicated to be partially homologous with the residues 195–214 of the hRSV fusion protein sequence derived from strain A2. These residues correspond to the α4-helix and α4–α5 loop within the prefusion RSV F structure. For all known structures of prefusion pneumovirus or paramyxovirus fusion proteins, the homologous sequence also corresponds to the equivalent α4-helix and α4–α5 loop, even if the residue numbering differs. To prevent overrepresentation from viral species which have multiple subtypes sequenced, we only included a single amino acid sequence from each species when the multiple subtypes were >90% identical throughout the residue range corresponding to 195–214 in hRSV F. However, if two subtypes within a single viral species differed by >10% in the residue range corresponding to 195–214 in hRSV F, then they were both included when performing sequence analysis and generating the sequence WebLogo. For example, there are four sequenced strains of human metapneumovirus F, but they are all mostly identical and hence only two representative sequences were included in the final WebLogo (strain A1 and B1). However, there are multiple distinct sequences for the different types of parainfluenza virus (PIV), and therefore all the distinct sequences are included separately when generating the WebLogo. See S1 Table for a full list of sequences used in the WebLogo. The WebLogo was generated using publicly available software at weblogo.berkley.edu.

### Accession numbers

The coordinates and structure factors for the F–RSD5-GL complex, the unbound AM22 Fab, and the F–AM22 complex, have been deposited in the Protein Data Bank (PDB) under accession codes 6DC3, 6DC4, and 6DC5, respectively.

## Supporting information

S1 Fig Alignment of VH and VK amino-acid sequences of parental RSD5 and RSD5-GL. Amino-acid sequence comparison of parental RSD5 (RSD5-WT) and its germline-reverted variant (RSD5-GL). Complementarity determining regions (CDRs) are highlighted in light blue according to IMGT. Dots indicate identical residues.
(TIF)

S2 Fig Comparison of the neutralization potency and binding kinetics of parental RSD5 with its germline-reverted variant RSD5-GL. (A) Neutralization of eleven RSV A and B strains by parental RSD5 (RSD5-WT) and RSD5-GL. IC_50_ values (ng/mL) were determined by immunofluorescence analysis and nonlinear fitting. The data with complete viral strain designations and numbers are reported in the Methods. Statistical significance was evaluated by Mann-Whitney U test. (B) SPR sensorgrams demonstrate that parental RSD5-WT has similar binding affinity and kinetics to RSD5-GL. The raw data is plotted as a black line and the fitted curve is shown as a thicker red line. Each binding curve has a 180 second association phase, followed by a 500 second dissociation phase. The equilibrium dissociation constant (*K*_D_) is shown directly above the sensorgram curves, while the association (*k*_a_) and dissociation (*k*_d_) rate constants are shown below the sensorgram.
(TIF)

S3 Fig AM22, RSD5-GL, and D25 do not bind to postfusion RSV F. SPR sensorgrams demonstrate that AM22, RSD5-GL and D25 do not bind to postfusion RSV F derived from (A) subtype A or (B) subtype B. The raw data is plotted as a black line. Motavizumab (Mota) Fab is a conformation-independent antibody and is included as a positive control, and the fitted curve in this sensorgram is shown as a thicker red line. Each binding curve has a 180 second association phase, followed by a 500 second dissociation phase.
(TIF)

S4 Fig AM22 binds with decreased affinity to subtype A prefusion RSV F with a K209Q substitution. SPR sensorgram of AM22 Fab binding to subtype A prefusion RSV F with a K209Q substitution. The raw data is plotted as a black line and the fitted curve used to calculate the binding kinetics is plotted as a thicker red line. Each binding curve has a 180 second association phase, followed by a 500 second dissociation phase. The equilibrium dissociation constant (*K*_D_) is displayed immediately above the SPR curve. The association rate constant (*k*_a_) and dissociation rate constant (*k*_d_) are shown below the sensorgram.
(TIF)

S5 Fig Ensemble of site Ø conformations in previously reported crystal structures. Ribbon representations of the conformations adopted by prefusion RSV F site Ø when bound to neutralizing antibodies. Only site Ø is shown for clarity. The color legend indicates which prefusion RSV F–antibody complex corresponds to which ribbon representation.
(TIF)

S1 Table Fusion glycoprotein sequences from paramyxoviruses and pneumoviruses used to construct the WebLogo in Fig 5D. Amino acid sequences of the fusion glycoproteins used to construct the WebLogo in Fig 5D.
(DOCX)
