# Supplementary figures and images for: Alternative conformations of a major antigenic site on RSV F
Source: PLoS Pathog. 2019 Jul 15;15(7):e1007944. doi: 10.1371/journal.ppat.1007944 (PMC6658013; doi:10.1371/journal.ppat.1007944)

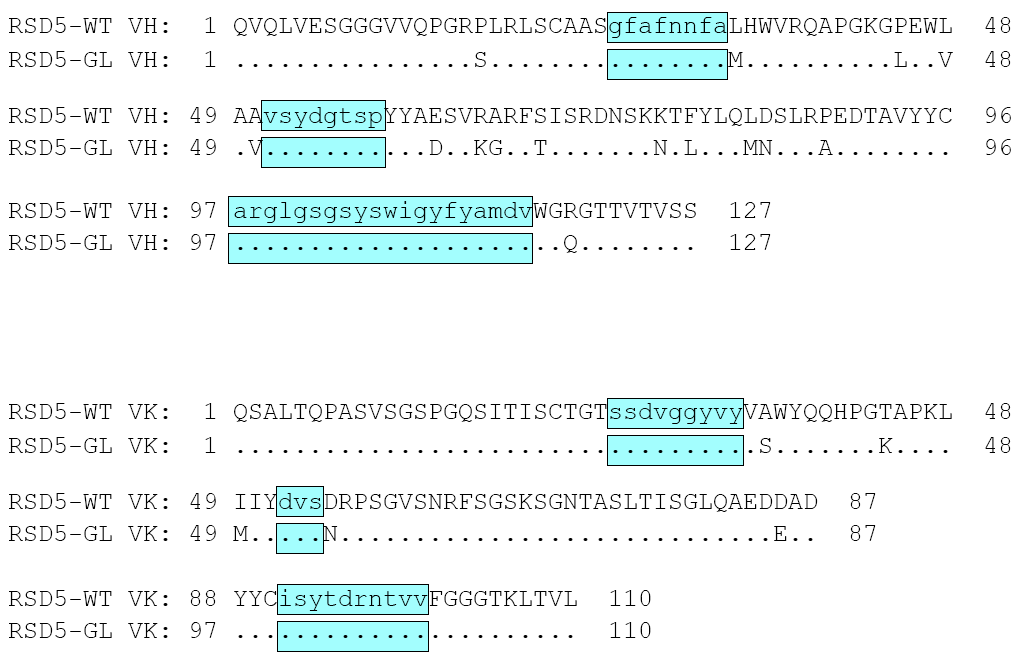

Supplement: S1 Fig — Amino-acid sequence comparison of parental RSD5 (RSD5-WT) and its germline-reverted variant (RSD5-GL). Complementarity determining regions (CDRs) are highlighted in light blue according to IMGT. Dots indicate identical residues. (TIF) [file ppat.1007944.s001.tif]

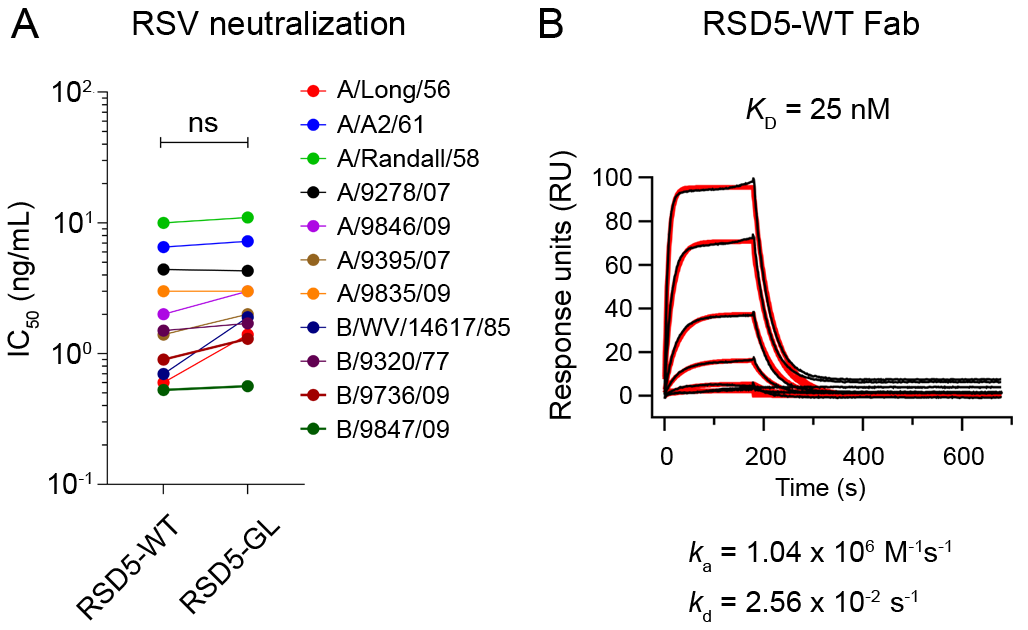

Supplement: S2 Fig — (A) Neutralization of eleven RSV A and B strains by parental RSD5 (RSD5-WT) and RSD5-GL. IC50 values (ng/mL) were determined by immunofluorescence analysis and nonlinear fitting. The data with complete viral strain designations and numbers are reported in the Methods. Statistical significance was evaluated by Mann-Whitney U test. (B) SPR sensorgrams demonstrate that parental RSD5-WT has similar binding affinity and kinetics to RSD5-GL. The raw data is plotted as a black line and the fitted curve is shown as a thicker red line. Each binding curve has a 180 second association phase, followed by a 500 second dissociation phase. The equilibrium dissociation constant (KD) is shown directly above the sensorgram curves, while the association (ka) and dissociation (kd) rate constants are shown below the sensorgram. (TIF) [file ppat.1007944.s002.tif]

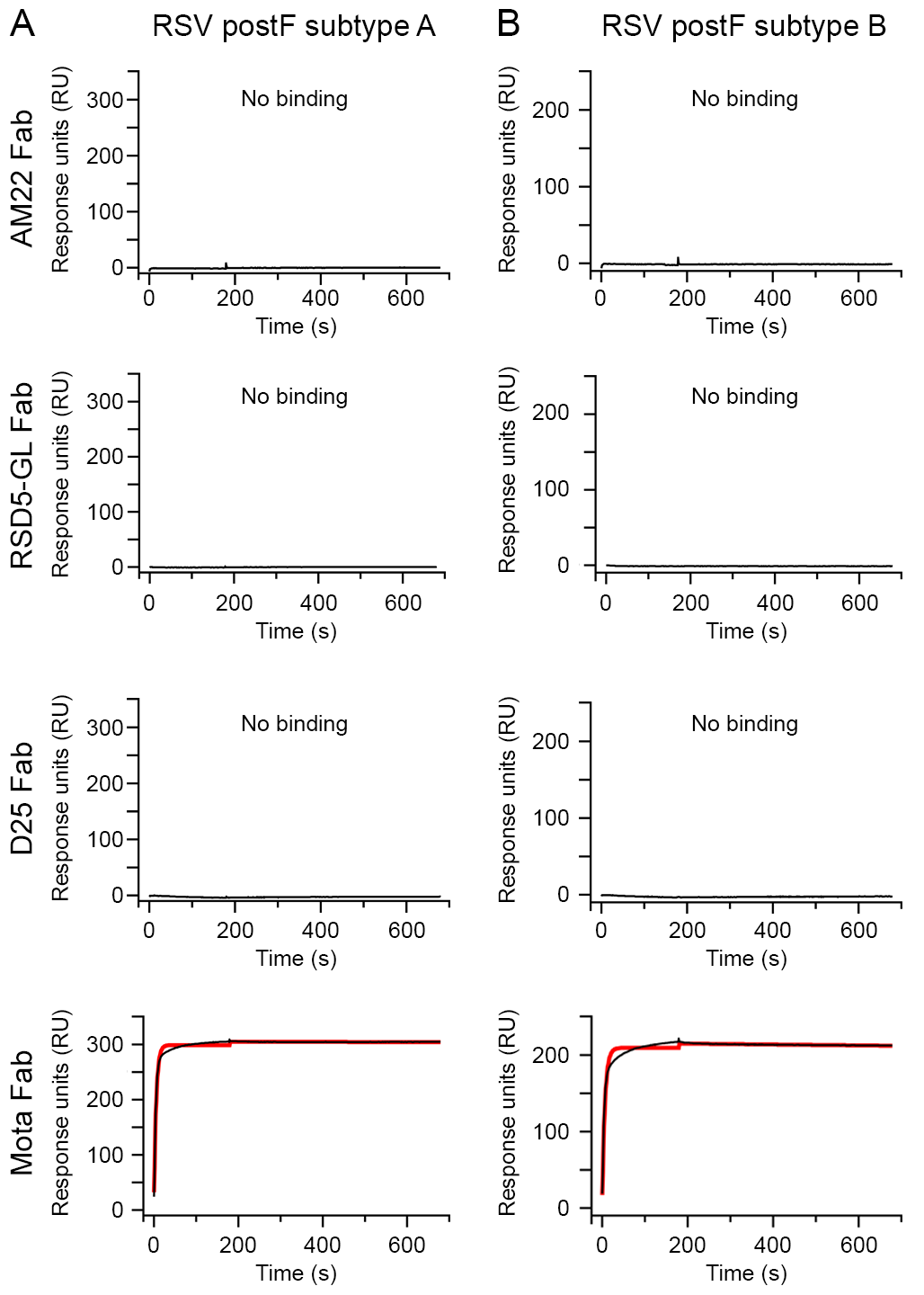

Supplement: S3 Fig — SPR sensorgrams demonstrate that AM22, RSD5-GL and D25 do not bind to postfusion RSV F derived from (A) subtype A or (B) subtype B. The raw data is plotted as a black line. Motavizumab (Mota) Fab is a conformation-independent antibody and is included as a positive control, and the fitted curve in this sensorgram is shown as a thicker red line. Each binding curve has a 180 second association phase, followed by a 500 second dissociation phase. (TIF) [file ppat.1007944.s003.tif]

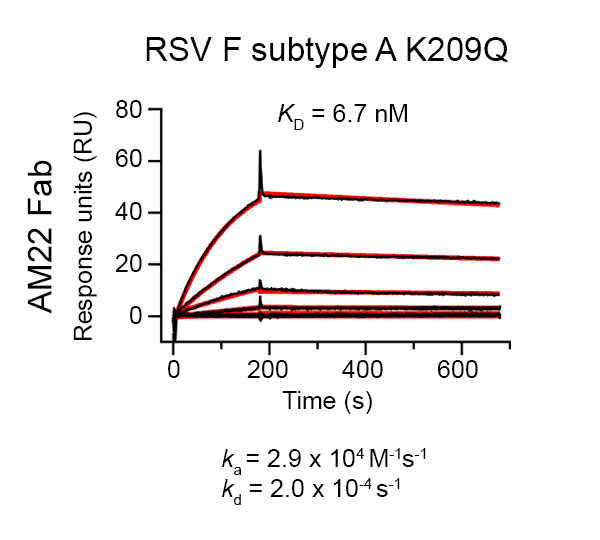

Supplement: S4 Fig — SPR sensorgram of AM22 Fab binding to subtype A prefusion RSV F with a K209Q substitution. The raw data is plotted as a black line and the fitted curve used to calculate the binding kinetics is plotted as a thicker red line. Each binding curve has a 180 second association phase, followed by a 500 second dissociation phase. The equilibrium dissociation constant (KD) is displayed immediately above the SPR curve. The association rate constant (ka) and dissociation rate constant (kd) are shown below the sensorgram. (TIF) [file ppat.1007944.s004.tif]

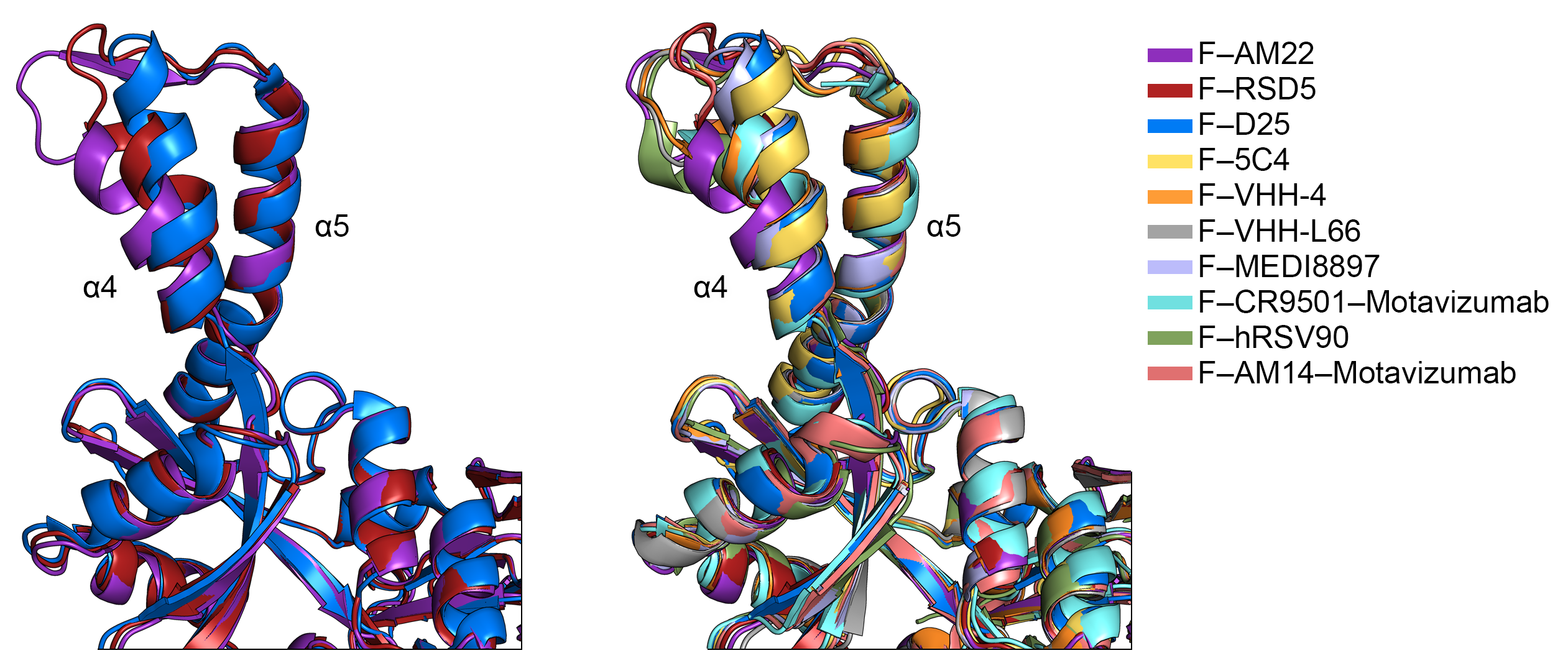

Supplement: S5 Fig — Ribbon representations of the conformations adopted by prefusion RSV F site Ø when bound to neutralizing antibodies. Only site Ø is shown for clarity. The color legend indicates which prefusion RSV F–antibody complex corresponds to which ribbon representation. (TIF) [file ppat.1007944.s005.tif]
