# Supplementary material for: Alternative conformations of a major antigenic site on RSV F
Source: PLoS Pathog. 2019 Jul 15;15(7):e1007944. doi: 10.1371/journal.ppat.1007944 (PMC6658013; doi:10.1371/journal.ppat.1007944)
Supplement: S1 Table — Amino acid sequences of the fusion glycoproteins used to construct the WebLogo in Fig 5D. (DOCX) [file ppat.1007944.s006.docx]

**S1 Table: Fusion glycoprotein sequences from paramyxoviruses and pneumoviruses used to construct the WebLogo in Fig 5D**

| **Organism** | **UniProtKB Accession #** | **Sequence** |
| --- | --- | --- |
| Human respiratory syncytial virus A  (strain A2) | P03420 | 195-LKNYIDKQLLPIVNKQSCSI-214 |
| Human respiratory syncytial virus B  (strain 18537) | P13843 | 195-LKNYINNRLLPIVNQQSCRI-214 |
| Bovine respiratory syncytial virus  (strain A51908) | P29791 | 195-LKNYIDKELLPKVNNHDCRI-214 |
| Human metapneumovirus A1  (strain NL/1/00) | Q91F55 | 165-LKDFVSKNLTRAINKNKCDI-184 |
| Human metapneumovirus B1  (strain NL/1/99) | Q6W8S5 | 165-LKEFVSKNLTSAINRNKCDI-184 |
| Murine pneumonia virus (strain 15) | P35949 | 160-LKNFISKELLPKINRVSCDV-179 |
| Newcastle disease virus (strain B1/48) | Q9DLD4 | 182-MQQFVNDQFNKTAQELDCIK-201 |
| Mumps virus (Miyahara vaccine) | P11236 | 168-IQDHINTIMNTQLNNMSCQI-187 |
| Measles virus (strain Ichinose-B95a) | Q786F3 | 178-VQDYINNELIPSMNQLSCDL-197 |
| Hendra virus | O89342 | 175-LQDYINTNLVPTIDQISCKQ-194 |
| Nipah virus | Q9IH63 | 175-LQDYINTNLVPTIDKISCKQ-194 |
| Sendai virus (strain Ohita) | O57295 | 182-LQDFVNDEIKPAISELGCET-201 |
| Reptilian ferlavirus (FDLV) | Q6YIS1 | 176-IQDHINSVINPALNQLGCDV-195 |
| Atlantic salmon paramyxovirus  (isolate -/Norway/Yrkje371/1995) | B2BX76 | 177-LQDQVNTNIIPAINTLGCTA-196 |
| Human parainfluenza virus 1  (strain C39) | P12605 | 178-LQDFVNNEIRPAIGELRCET-197 |
| Human parainfluenza virus 2 | P25467 | 172-IQDHINGAIVNGITSASCRA-191 |
| Human parainfluenza virus 3  (strain NIH 47885) | P06828 | 175-VQDYVNKEIVPSIARLGCEA-194 |
| Human parainfluenza virus 4a  (strain M-25) | D7US59 | 169-IQNQINTIIQPAIDQINCQI-188 |
| Human parainfluenza virus 4b  (strain QLD-01) | A0A097F1I2 | 169-IQNQINTVIQPAIDRIDCQI-188 |
| Parainfluenza virus 5 (strain W3) | P04849 | 168-VQDHINSVVSPAITAANCKA-187 |
| Bovine parainfluenza virus 3 | P09990 | 175-VQDYVNNEIVPSITRLGCEA-194 |
| Canine distemper virus  (CDV strain Onderstepoort) | P12569 | 290-VQDYVNNELVPAMQHMSCEL-309 |
| Simian virus 41 (SV41) | P25181 | 175-VQDHVNGVLASGLTEANCRS-194 |
| Menangle virus | K9MY70 | 171-IQNQVNEVINPAINKLSCEV-190 |
| Rinderpest virus (strain RBOK) | P41356 | 174-VQDYINNELVPAMGQLSCDI-193 |
